# Planning for pre-exposure prophylaxis to prevent HIV transmission: challenges and opportunities

**DOI:** 10.1186/1758-2652-13-24

**Published:** 2010-07-12

**Authors:** Susan C Kim, Stephen Becker, Carl Dieffenbach, Blair S Hanewall, Catherine Hankins, Ying-Ru Lo, John W Mellors, Kevin O'Reilly, Lynn Paxton, Jason S Roffenbender, Mitchell Warren, Peter Piot, Mark R Dybul

**Affiliations:** 1 O'Neill Institute for National and Global Health Law, Georgetown University, Washington DC, USA; 2 Bill & Melinda Gates Foundation, Seattle, Washington, USA; 3 National Institutes of Allergy and Infectious Diseases, Washington DC, USA; 4 Joint United Nations Programme on HIV/AIDS, Geneva, Switzerland; 5 World Health Organization, Geneva, Switzerland; 6 University of Pittsburgh, Pittsburgh, USA; 7 Centers for Disease Control and Prevention, Washington DC, USA; 8 AIDS Vaccine Advocacy Coalition, New York, USA; 9 Institute for Global Health, Imperial College London, London, UK; 10 George W Bush Institute, Dallas, TX, USA

## Abstract

There are currently several ongoing or planned trials evaluating the efficacy of pre-exposure prophylaxis (PrEP) as a preventative approach to reducing the transmission of HIV. PrEP may prove ineffective, demonstrate partial efficacy, or show high efficacy and have the potential to reduce HIV infection in a significant way. However, in addition to the trial results, it is important that issues related to delivery, implementation and further research are also discussed. As a part of the ongoing discussion, in June 2009, the Bill & Melinda Gates Foundation sponsored a *Planning for PrEP *conference with stakeholders to review expected trial results, outline responsible educational approaches, and develop potential delivery and implementation strategies. The conference reinforced the need for continued and sustained dialogue to identify where PrEP implementation may fit best within an integrated HIV prevention package. This paper identifies the key action points that emerged from the *Planning for PrEP *meeting.

## Introduction

Recent data suggest that current efforts to prevent and treat HIV are beginning to yield results. Significant expansion of antiretroviral therapy has led to decreased mortality. There has been some stabilization or decline in new HIV infections across several countries in sub-Saharan Africa, which is home to 67% of all people living with HIV [1]. Trend data indicate that there were 400,000 fewer new infections in that region in 2008 than there were in 2001 [2]. In South Africa specifically, there were 40% to 60% reductions in new HIV infections among youth over a five-year period ending in 2008 [3].

Despite these promising developments, much work remains. Overall HIV prevalence is unacceptably high and continues to rise in parts of the world. Approximately 33 million people were living with HIV and 2.7 million were newly infected in 2007 [1]. In eastern Europe and Asia, HIV disproportionately affects men who have sex with men (MSM), injecting drug users (IDUs) and sex workers [1].

Although behavioural interventions are important, and structural initiatives are needed to address the underlying determinants of vulnerability to HIV, technology can also provide needed additions to the prevention arsenal. Advances in the understanding of the pathogenesis of HIV have led to more sophisticated research on prevention strategies. Research has shown that early in infection, HIV targets and destroys the cells of the immune system that are likely best adapted to prevent establishment and control progression of disease [4]. It is therefore important to intervene before infection is established and pursue all avenues to develop a well-planned package of "combination prevention". This package should include effective and complementary modalities to decrease rates of HIV transmission to the greatest extent possible [5,6].

One technological prevention option currently under study that could be of great value is pre-exposure prophylaxis (PrEP). PrEP is a strategy for HIV-negative individuals to reduce or prevent their risk of infection by taking oral antiretroviral drugs used for HIV treatment or by applying microbicides containing the active antiretroviral agent.

In June 2009, as part of the ongoing discussion surrounding PrEP, the Bill & Melinda Gates Foundation (BMGF) sponsored a *Planning for PrEP *conference, which was attended by a number of individuals and organizations currently working in areas relevant to PrEP. The purpose of this meeting was to discuss the expected results of the ongoing PrEP clinical trials, critically examine the relevant policy and research issues likely to emerge, and advocate for coordinated and collective action among the various participants to address these issues. This paper outlines the key action points that emerged from the conference.

## Main text

### PrEP and HIV

The general principle underlying PrEP is straightforward: drugs that are available as treatment can be used to prevent infection among persons highly exposed to a pathogen or those who are otherwise more vulnerable to infection. Chloroquine was used to both treat malaria and to prevent it among persons travelling to malarious regions, and isoniazid is still used as prophylaxis in high-risk groups and as part of the treatment regimen for tuberculosis. Looking generally at the current epidemiology of the HIV epidemic (serodiscordant couples in Africa and marginalized populations), PrEP may be an appropriate targeted prevention strategy.

Several completed pre-clinical studies in different animal models have shown promise. Macaques treated daily with emtricitabine (FTC) alone were less likely to become infected following rectal exposure to a simian version of HIV (SHIV) than untreated animals. Macaques treated with oral FTC and tenofovir disoproxil fumarate (TDF) at doses similar to a human equivalent dose had an even smaller rate of seroconversion. In addition, macaques injected daily with two antiretroviral drugs, FTC and TDF, at high doses were completely protected from infection [7].

Human clinical trials to evaluate PrEP as a strategy to prevent the transmission of HIV began in 2004. In the intervention arms of the current clinical studies, HIV-negative adults receive antiretroviral products formulated as pills, gels, etc., that block HIV replication during periods of HIV exposure as prophylaxis against infection [8,9]. As presently studied, "periods of exposure" require continuous use of the product (or in the case of PrEP gels, are coitally dependent).

**Figure 1:**
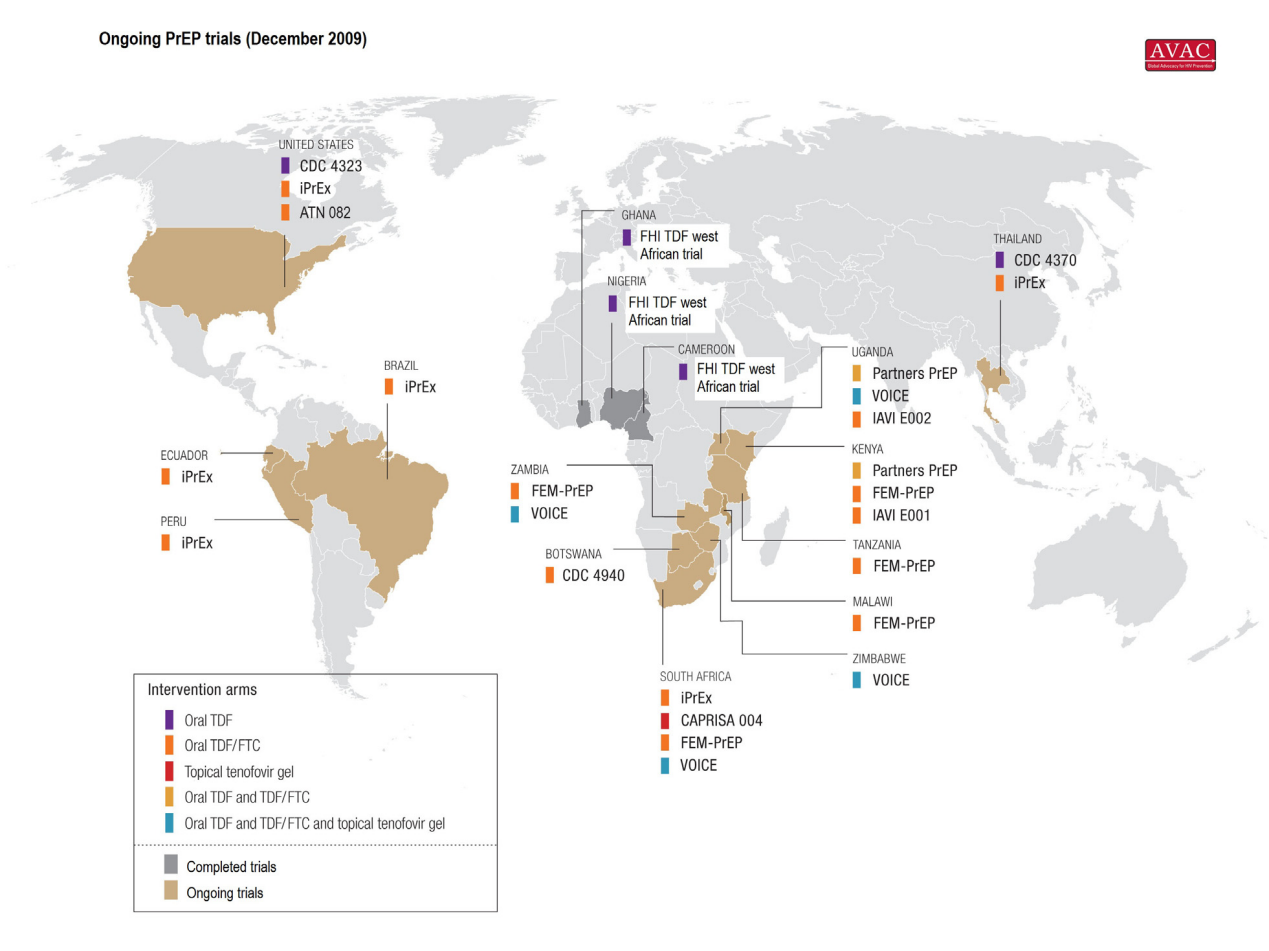
**PrEP trials map - December 2009**. PrEP-specific map gives a view of ongoing and completed PrEP trials worldwide. (Map: AIDS Vaccine Advocacy Coalition)

As of March 2010, there are 10 ongoing PrEP clinical trials in humans using TDF, TDF gel, or TDF/FTC [10] (see Additional file 1). The studies encompass diverse populations including: injecting drug users (IDUs) in Thailand; men who have sex with men (MSM) in South America, Africa, Thailand and the United States; serodiscordant heterosexual couples in Kenya and Uganda; and women who are at higher risk of becoming HIV infected through sexual intercourse in eastern and southern Africa [11] (see Figure 1). These studies are primarily funded by the US National Institutes of Health (NIH), the US Centers for Disease Control and Prevention (CDC), the Bill & Melinda Gates Foundation (BMGF), and the US Agency for International Development (USAID). Gilead Sciences manufactures both TDF and TDF/FTC and provides the drugs for these trials.

Results from the US-based Extended Safety Trial (CDC 4323) are expected in 2010 [10]. The first efficacy trial results should also be available by the end of 2010, and could be reported earlier based on recommendations made by the independent Data Safety and Monitoring Boards (e.g., the MSM study and the Thai IDU study both expect to have final results by 2010). Therefore, it is important for public health decision makers to prepare a collective, coordinated and rational response to the results of the PrEP trials [12,13]. As part of ongoing efforts, the BMGF sponsored a *Planning for PrEP *conference in June 2009.

### Planning for PrEP

The objective of the one-day meeting was to gather stakeholders to discuss what can realistically be expected from the ongoing PrEP clinical trials, and examine how those expected clinical data points can be most rapidly translated into public health impact. The participants concluded that the most effective strategy would be to develop a "proof of deliverability" pathway to accompany the ongoing clinical proof of concept studies. This would avoid any unnecessary delay in implementation and delivery of PrEP, should the studies show efficacy. To achieve this, there must be diversity of expertise, but also unity of purpose among the various stakeholders. Sustained communication, collaboration and collective action will be required.

This paper outlines the key action points that emerged from the *Planning for PrEP *meeting. The findings represent the most salient themes discussed. Although there is some overlap between issues, they form a broad picture and illustrate the necessary integration of research and policy to develop a comprehensive implementation framework. They also reflect the importance of the overall goal of developing concurrent clinical proof of concept and proof of deliverability strategies. The June 2009 consultation was the launching point of a multi-institutional effort to examine the major policy, regulatory, delivery, programme implementation and user-perspective issues surrounding PrEP in greater detail.

### Key action points

#### 1. Show proof of deliverability

Once proof of concept for PrEP has been obtained, it will be essential to establish proof of deliverability. "Proof of deliverability" examines whether PrEP can be delivered. To determine this, an overall delivery and implementation framework that demonstrates the feasibility of PrEP in different cultural, ethical, legal and political contexts will need to be developed. Delivery and implementation of any intervention should be tailored in ways that are best suited to the local, national and regional epidemics, and to individual user preferences that recognize social and cultural norms and practices. The research to determine proof of deliverability will therefore need to be customized for each target population.

This analysis will include modelling on cost effectiveness and market acceptability of PrEP in the various target populations. It must also assess the resources required for optimal delivery of PrEP. This analysis must include the human, infrastructure and financial requirements necessary, globally and within countries. The design and execution of this research and analysis should begin while clinical trials are underway to understand the challenges and opportunities of PrEP and to develop strategies with the greatest likelihood of success [14]. Identifying these challenges will likely have applications in other HIV prevention contexts.

##### a. Model costs and benefits

Because each country (and different areas within countries) has different HIV epidemics, it will be important for decision makers to have data on the costs and benefits of delivering PrEP that are epidemic-specific. Although some analysis has already been conducted, the existing modelling on PrEP is limited and does not provide a sufficiently comprehensive analysis to fully understand the implications of the intervention.

To support proof of deliverability, more comprehensive and sophisticated modelling should be done now to examine for which populations (e.g., sex workers, MSM and IDUs, and the highest risk groups within these groups), in what scenarios (e.g., concentrated epidemic, generalized epidemic), and for what levels of effectiveness PrEP would have the greatest impact at the lowest cost. Models should also include variables that address HIV re-testing and the development of resistance to TDF and FTC in both the persons who will use PrEP and the general population, as well as the related costs of later antiretroviral therapy for those who are infected with a resistant virus. Modelling should determine costs per HIV infection averted for a variety of service delivery strategies, target populations, providers and speed of scale up. This research should underpin the development of a user-friendly decision makers' programme planning tool.

There is also an urgent need for modelling on products other than those currently being used in trials to inform new areas of clinical study. Cost-benefit analysis of other products that includes the emergence of drug resistance is necessary. It is important that this analysis consider a broad spectrum of outcomes related to PrEP. For example, researchers should evaluate the costs of delivery by qualified clinicians, as well as the savings in human and financial resources to a health sector from a significant reduction in HIV infections, and consider indirect costs saved.

##### b. Conduct targeted market research

For a potential new prevention option, such as PrEP, public understanding and perception will be a critical element for its successful introduction in a community. It is vital to assess the initial level of understanding and perceptions of PrEP among stakeholders and different consumer segments to better inform policy development. It will be necessary to identify issues that constituencies may have in terms of introducing PrEP in their communities.

Targeted market research of these constituencies will draw on current knowledge in marketing, strategy and organizational behaviour to identify, evaluate and address potential concerns and expectations by stakeholders and consumers across individual communities and segments. Such research is needed to enhance the chances of adoption of PrEP in the future and the development and adoption of potential next-generation products. This research will shed light on the relative importance of a number of marketing factors (distribution, communication, patient preferences, interaction with existing health campaigns, etc.) that can support or hinder the successful introduction of PrEP in a community.

##### c. Establish regulatory pathways

Rapid movement through regulatory pathways will be an integral component of effective and efficient delivery of PrEP. There are several outstanding issues related to regulatory review and approval. The demonstration of clear safety of PrEP as a prevention strategy will be integral to this review. Additionally, the antiretroviral drugs currently under study for use in PrEP are approved for treatment only, not for prevention. To use these drugs for prevention, a new regulatory indication is needed, or they would be used off-label. Decisions made by the World Health Organization (WHO) and leading national regulatory agencies, for example, the US Food and Drug Administration (FDA) and the European Medicines Agency (EMEA), on labelling requirements will likely have a significant impact on national decision makers and global funders, including the Global Fund to Fight AIDS, Tuberculosis and Malaria and the US President's Emergency Plan for AIDS Relief (PEPFAR).

Each country and global funder of HIV prevention programmes will need to develop an agreement on a regulatory framework. It is essential that this dialogue begins now because the process to establish these pathways is administratively and politically cumbersome and will require collaboration among a number of stakeholders, including drug companies (both innovator and generic), national regulatory authorities, normative bodies, such as WHO and the Joint United Nations Programme on HIV/AIDS (UNAIDS), and funders, such as the BMGF and PEPFAR.

It is possible that the use of antiretroviral products currently approved for treatment will not require regulatory approval for off-label use as prevention in order to be financed by major funders. For example, nevirapine has not been approved by regulatory authorities for use in prevention of mother to child transmission (PMTCT) programmes, but is funded because of broad support and guidance from national and global normative bodies, such as WHO [15]. Pursuing regulatory approval could significantly and unnecessarily slow down scale up of PrEP. It is important that there be a full discussion of the advantages, disadvantages and necessity of pursuing various regulatory pathways for PrEP. Global consultations are being planned in 2010 to examine these issues.

##### d. Develop an implementation framework

In order to translate any clinical trial result into public health impact, it is important that global, regional and national dialogues take place to articulate components of an implementation framework. A global framework should be applicable to national strategies and, therefore, should include parameters that will facilitate the alignment of the PrEP research agenda with the varied interests of stakeholders, which include policymakers, service delivery providers, those living with HIV, researchers and advocates.

There are several important components that must be integrated into an effective implementation framework. It should expand upon the lessons learned from the scale up of other proven interventions, such as PMTCT and male circumcision. It should identify entry points and cross linkages for the successful rollout of PrEP in different user populations. An integral component of an implementation strategy will be to assess the drivers of the epidemic in a community or nation and to programme the appropriate mix of interventions that are likely to have the greatest impact on HIV prevention. Mathematical modelling can be helpful in making such decisions.

The framework should also address such issues as programme design in different resource settings targeting specific user populations, facilitation of rapid rollout, integration of counselling to maximize adherence and minimize risk practices, and ensuring regular HIV re-testing to optimize impact and minimize resistance. Early lessons learned for implementation could be provided by post-trial access programmes following the current clinical trials. Funders of those studies have agreed, in principle, to support such programmes.

It is important that the dialogue increases commensurate to the research and endeavours to optimize the chances for successful global and national level rollout of PrEP. A successful implementation framework will require a combination of many individual preparatory steps that require input and action by the largest possible range of stakeholders.

#### 2. Importance of coordination and collaboration

The success of any comprehensive global prevention strategy will be contingent on the strength of coordination and collaboration between relevant stakeholders. These include potential user populations, clinical researchers, normative bodies, funders, regulatory agencies, drug companies, policymakers and advocacy organizations. An important aspect of global coordination and collaboration will be the ongoing collection of data, assessments, and monitoring and evaluation to develop and share lessons learned.

The current global economic downturn and shifting of funding priorities away from global health also makes it essential to optimize resources in order to advance the field and minimize duplication in a timely manner. Regardless of what the clinical trials show, without greater cohesion and interaction in the field, successful delivery and implementation of PrEP will be elusive.

#### 3. Develop effective communication strategies

Highly effective interventions with well-designed implementation strategies can be quickly shelved unless there is adequate understanding and acceptance in a community. A comprehensive communication effort is an imperative with PrEP. Several organizations have already begun to provide information on the data that will become available as trials are reported, including the AIDS Vaccine Advocacy Coalition (AVAC), the CDC, WHO and UNAIDS. A cross-trial PrEP Communications Working Group is already active, but a more expansive effort will be required in the coming months to ensure that potential users, policymakers and other stakeholders are given detailed information to make choices and decisions about PrEP in their respective settings.

Open and honest dialogue should occur now across affected communities so that a common understanding can be reached and appropriate messages developed. This dialogue should incorporate such issues as the complexity of the clinical trials, costs and benefits of interventions (including the risks of resistance), the difficult issue of providing antiretroviral therapy for prevention in settings with unmet treatment needs, and other potential problems.

It will also be important to convey that delivery and implementation of PrEP will vary by country. While it is essential that countries and communities learn from each other, decisions made on the adoption of PrEP in one country or setting should not unduly influence other countries. It will be essential to closely evaluate communications issues and acceptability of PrEP in early-adopter environments to share lessons learned as global scale up occurs.

#### 4. Importance of country ownership

All health is local, and the key to any successful intervention is country leadership and ownership. There is no single global HIV epidemic. There are many HIV epidemics, often within the same country. Preventing HIV is complex and involves many social issues. Technological interventions are influenced by the cultural and political environments in which they are used.

While certain issues related to PrEP will be constant across borders, there will be particular issues in different country contexts. Understanding the expectations and goals of affected communities in each country will be an essential component of successful rollout of PrEP. Decision makers in each country will wrestle with the data that are available from the trials and develop combination prevention strategies that are tailored to their specific needs and to their specific epidemics. A central issue in low- and middle-income countries will be the acceptability of introducing antiretroviral drugs for use in prevention when there are unmet needs in treatment. Policymakers will be confronted with the ethical implications of how to divide a limited pool of resources for drugs between two separate groups (i.e., people who are uninfected versus HIV-positive people). This issue will likely be the most problematic for policymakers, advocates and other stakeholders.

Now is the time to begin the complex discussions and to establish a long-term strategy to engage multiple communities in each country where PrEP research is taking place or planned. These should include potential users of PrEP, health care providers, civil society, policymakers, regulators, the media, people living with HIV and other stakeholders. Discussions should address the research studies' trial designs and potential findings, policy issues, and the challenges and opportunities of delivery and implementation. It will also be essential to explore what additional information is needed by these communities and to begin new studies to provide data to help resolve complicated issues.

Consultations should begin immediately so that decision makers will be in the best position to act when data become available. A particular opportunity to convene and discuss exists in countries where PrEP trials are currently underway. Lessons learned from those presumed early adopters could be useful to inform discussions in other countries and in the global arena. Preparations for these in-country and regional consultations are currently underway.

#### 5. Role of normative bodies

Although national normative groups and health authorities will be the ultimate arbiters for the availability of PrEP in their countries, WHO and UNAIDS will have an important role in setting global norms and standards. WHO follows the GRADE approach to develop new recommendations, which involves systematic reviews and quality assessments of evidence, risk/benefit analyses, expert and community consultations, and a number of Guideline Development Meetings with designated constituencies [16]. This approach considers operational feasibility and cost of the proposed interventions. Careful balancing of the risks and benefits, as well as costs in light of national decision making for use of antiretrovirals not only for treatment but also for prevention, will be critical. Subsequent to consultations on the scope and questions covered in the guidelines, the process usually takes at least nine and 12 months to complete.

At the global, regional and in-country levels, WHO and UNAIDS will play an instrumental role in convening stakeholders to identify implementation challenges, ethical issues and solutions and propose new avenues for operational research and implementation strategies. As their work with male circumcision demonstrated, the process is most effective when there is a strong collaboration with a wide spectrum of stakeholders from researchers to community-based organizations [17].

#### 6. Clinical trial results will only be the beginning

The ongoing clinical trials will provide information on efficacy, but they will only be the beginning to an understanding of how PrEP might contribute to HIV prevention. The studies are powered at 50% to 60% drug efficacy. Depending on the strength of the data, including the level of statistical significance, one trial could be sufficient to establish proof of concept to promulgate normative guidelines and/or obtain regulatory approval. However, if efficacy is weak, or if there are significant policy issues, in particular, applying results across populations, additional trials will likely be needed (one trial with robust and compelling data with a p value of 0.001, or two trials with p values of 0.05). It should also be noted that efficacy in a trial setting does not show proof of deliverability in non-trial settings.

The studies will establish the degree to which PrEP prevents the transmission of HIV and is viable as proof of concept in persons at high risk of infection, including MSM, IDUs, serodiscordant couples and others at risk of heterosexual transmission. These studies will also provide important data on the short-term safety and tolerability of the agents used in HIV seronegative individuals.

There will be limited information about other important matters. The trials will provide some preliminary information on adherence, but it will likely be overestimated because the data will be based on self reports. Adherence is also likely to be overestimated because study participants are closely monitored and evaluated, and there is usually better adherence in a trial context. There may also be some data on the risks of drug resistance among those who become infected while receiving PrEP in the context of a closely monitored clinical trial, but there will be no information on the risks to the population due to those individuals transmitting drug resistant-HIV to others. Also, while there may be some initial data on the role of PrEP and possible risk compensation among those who receive it, these trials will not sufficiently address the issue of risk compensation.

The level of efficacy shown from these trials will establish priorities for future study and public health decision making. Per-protocol analysis will be instrumental in assessing the efficacy of the intervention physiologically, though intention-to-treat analysis will begin to address the equally important issue of adherence. With suboptimal adherence, which may vary significantly in the different populations participating in the current studies, the effectiveness of the intervention is limited. Studies evaluating the effectiveness of intermittent or episodic dosing would contribute to our knowledge of the intervention, specifically the cost effectiveness of different approaches to prophylactic administration of antiretroviral medicines.

The ongoing studies exclude groups that may eventually benefit from the intervention, such as adolescents, pregnant or nursing women, and those with hepatitis B infection or renal or hepatic disease. A more accurate picture of the effectiveness of PrEP in these populations, which are also at higher risk of HIV infection, will require additional study. Future trials may be more logistically difficult to conduct once proof of principle is established as the use of placebo may no longer be ethical. The current guidance on standard of prevention in the research context states that new prevention modalities should be introduced when they are scientifically validated or approved by national authorities [18]. This is complicated by the fact that there is no uniform process for determining when to introduce a new modality, although negotiations among all research stakeholders are recommended to determine the appropriate standard of prevention for a specific trial. The ability to fund interventions and the feasibility of trial conduct also play a role in this determination.

It is important to note that each trial has unique characteristics and will provide additive data on the different methods of PrEP intervention, as well as the varied populations for which intervention may be most effective. It will be essential to continue all currently ongoing trials. The results of these initial studies may lead to additional studies that address new research questions exploring proof of concept and proof of deliverability. As the AIDS Vaccine Advocacy Coalition (AVAC) has emphasized:

While it is impossible to predict the future, it is highly likely--based on experiences with other biomedical HIV prevention strategies--that other [PrEP] efficacy trials would continue even if a single trial showed benefit. It is critical to answer questions about the safety, acceptability and efficacy of different product formulations and combinations, in different populations in which the routes of transmission differ. In addition, even if several of the ongoing efficacy trials find that PrEP is safe and effective in reducing HIV risk, there will still need to be additional research on long-term safety, use in pregnant women and adolescents, and to understand the potential effectiveness of other dosing and delivery strategies [19].

Although there will be important safety and efficacy information acquired from the current studies, there are several other significant issues that will not be addressed, including: the relative role of PrEP as part of a combination prevention strategy in different epidemiological settings (e.g., concentrated and generalized epidemics); the applicability of non-TDF-based regimens for PrEP; how PrEP will affect future disease progression and infectiousness in those that do become infected; and long-term safety. Safety will be essential to address policy issues, regulatory pathways and normative guidance.

#### 7. PrEP will not be a "magic bullet" that ends the HIV epidemic

No single intervention will be sufficient to prevent the transmission of HIV on a global scale. The current prevention landscape is comprised of several important but partially effective prevention interventions. Primary prevention approaches include behaviour change education and technological interventions, such as male and female condoms, male circumcision and needle syringe/safe injecting equipment distribution and opioid substitution therapy, as well as HIV testing and counselling. Secondary prevention strategies, the treatment of infected individuals, also include behavioural and technological interventions [20].

If trial results are favourable, showing both safety and efficacy, PrEP will be one additional option within a comprehensive, combination prevention package [21]. PrEP will not replace other effective interventions, eliminate the need to continue other preventative vaccine and microbicide research, or be an appropriate choice for everyone. For example, PrEP could be of benefit among discordant couples, which increasingly accounts for significant new infections in sub-Saharan Africa [22,23], although many argue that it is more relevant to treat the infected partner. In such relationships, there is regular sexual activity in which every episode is with an infected partner, but existing prevention mechanisms are not fully utilized. Condom use among regular partners is difficult [24]. Male circumcision, which is partially protective, has been shown to reduce the risk for the male partner in a serodiscordant, heterosexual couple [25]. These high-risk populations will likely require multiple interventions to have the greatest possible preventative impact at both individual and population levels.

It is important to note that the clinical trials may show partial efficacy of PrEP. Delivery and implementation of PrEP will need to be carefully considered with any result, but should studies show that PrEP's protective effectiveness falls by between 30% and 60%, there will be outstanding questions about the role and utility of PrEP in HIV prevention. If they choose to move forward with integrating PrEP into comprehensive HIV prevention programmes with partial efficacy, the public health community will have to develop appropriate guidelines and messaging.

As already discussed in greater detail, the use of PrEP in a given population will be a complicated issue to be determined by a diversity of stakeholders. Trial efficacy does not always translate directly into community effectiveness. Additional research and secondary analyses will be necessary to establish the clinical and public health relevance of PrEP and to determine the optimal use of PrEP.

Rather than creating undue emphasis on a single modality, PrEP should be considered one option in a larger prevention arsenal. This highlights the need for greater focus on how best to design and execute combination HIV prevention interventions that include technological interventions, e.g., for prevention of sexual transmission of HIV (condoms, male circumcision and PrEP), and behavioural interventions that target sexual behaviour and behaviour related to use of available technologies. The appropriate balance between the various interventions in a community or nation would be informed and directed by the drivers of the epidemic in the specific environment.

### Moving forward: next steps

Guided by the key action points, the meeting participants outlined several next steps to address and respond to identified knowledge gaps. Specific activities were then developed to facilitate the overall discussion on the delivery and implementation of PrEP. A necessary outcome over the next 12 to 18 months will be to develop goals and actions with an accountability framework. These coordinated and collaborative actions among the various stakeholders will help quickly identify potential solutions and limit delays in scaling up a potentially life-saving intervention.

### PrEP Steering Committee

A global PrEP Steering Committee will be established to facilitate the coordination of interested parties. The committee will be chaired by WHO and UNAIDS with representation from other organizations. As part of its responsibilities, the PrEP Steering Committee will convene periodic stakeholder consultations and will establish and coordinate working groups that are charged with examining specific issues.

### Working groups

To support the work of the steering committee and coordinate stakeholders working on PrEP, several working groups will sew together the threads of an effective scale up of PrEP should the current studies demonstrate a prevention effect. The working groups will address questions related to the current trials and implementation and delivery, including an effort to use implementation issues to inform an expanded research agenda. Four working groups are currently active: the Scientific Working Group, convened by the CDC; the Clinical Trialists Working Group, under the auspices of the Forum for Collaborative HIV Research; the Communications Working Group, convened by AVAC; and the Delivery Working Group, chaired by WHO, UNAIDS, Imperial College London and Georgetown University.

The various working groups are charged as follows. The Scientific Working Group examines issues related to the overall PrEP research agenda and develops areas for further study. The Clinical Trialists Working Group discusses operational issues specific to the ongoing clinical trials. The Communications Working Group supports the development and implementation of a comprehensive strategy for improving communication flow, stakeholder outreach and media relations. Finally, the Delivery Working Group focuses on issues relevant to the delivery and implementation of PrEP should the clinical studies show efficacy. These issues include specific policy, legal, cultural and ethical barriers to implementation of PrEP particular to various regions, analyses of regulatory issues, operational acceptability, feasibility and cost. Each working group will convene consultations and develop sub-groups as necessary to achieve their respective objectives.

The underlying goal of all working groups is to enhance coordination and collaboration (see Key Action Point: Importance of coordination and collaboration), and to provide decision makers, including normative bodies, with the requisite information they will need to develop and issue appropriate guidelines. In addition, knowledge acquired by the various groups will be appropriately coordinated and disseminated through open-access forums in order to have the widest application in the field. The BMGF has committed to fund these stakeholder and working group activities.

## Conclusions

While much progress has been made in HIV prevention and treatment, a sustainable strategy to turn the tide against HIV infection remains elusive. Combination prevention that includes biomedical, behavioural and structural interventions is required. The package of interventions will vary by the dynamics of the HIV epidemics in each country, or region of each country. Decision makers in each country must determine their optimal HIV prevention strategy and take the initiative in its design and implementation.

Animal studies have already suggested that PrEP could be an important weapon in HIV prevention, and current human clinical trials evaluating PrEP will begin reporting valuable data in 2010. Even if the data demonstrate a significant reduction in HIV infections, several scientific and policy questions will remain unanswered. Issues surrounding delivery, implementation, regulatory pathways, policy and communication will require sustained dialogue and resolutions to translate the research results into HIV infections averted and lives saved.

The overall message from the *Planning for PrEP *meeting was that there may be great promise in PrEP, but difficult issues must be addressed. Effective delivery and implementation of PrEP, as one component of an integrated HIV prevention package, will require participation and collaboration by stakeholders. While substantial work has already been done, especially with regard to the science of PrEP, a process is being developed by this group to push forward on several fronts. Other stakeholders working on PrEP issues or on issues related to PrEP are encouraged to engage in the described activities. This will help to ensure that everything that can be done to prepare for PrEP is being done in a coordinated, efficient, and inclusive fashion.

PrEP may prove ineffective. Or, it may turn out to be a unique and important new opportunity for the world to reduce HIV infection and change the course of the epidemic. PrEP research cannot be delayed unnecessarily for the sake of those at risk of contracting HIV. We also cannot wait for definitive clinical results before developing plans to utilize PrEP to maximize public health impact against the pandemic. Any delay in implementation of an effective prevention intervention will cost many lives. It is an ethical imperative that we act now to prepare the path to timely implementation.

## List of abbreviations used

The following are abbreviations found in the paper: AVAC: AIDS Vaccine Advocacy Coalition; BMGF: Bill & Melinda Gates Foundation; CDC: United States Centers for Disease Control and Prevention; FTC: emtricitabine; IDU: injecting drug user; MSM: men who have sex with men; NIH: United States National Institutes of Health; PrEP: pre-exposure prophylaxis; SHIV: simean/human immunodeficiency virus; TDF: tenofovir disoproxil fumarate; UNAIDS: Joint United Nations Programme on HIV/AIDS; and WHO: World Health Organization.

## Competing interests

The *Planning for PrEP *conference described in this paper was funded by the Bill & Melinda Gates Foundation. BMGF also supports some of the ongoing activities described. The authors declare that they have no competing interests.

## Authors' contributions

SK coordinated the drafting of this paper, supported by JR. SK, SB, CD, BH, CH, YRL, JM, KOR, LP, JR, MW, PP, and MD wrote, reviewed, and edited the manuscript. All authors read and approved the final manuscript.

## Supplementary Material

Additional file 1 **PrEP trials table - March 2010**. Table contains information about ongoing trials of oral PrEP and topical microbicides. (Table: AIDS Vaccine Advocacy Coalition) Click here for file
