# Supplementary figures and images for: Planning for pre-exposure prophylaxis to prevent HIV transmission: challenges and opportunities
Source: J Int AIDS Soc. 2010 Jul 12;13:24. doi: 10.1186/1758-2652-13-24 (PMC2914050; doi:10.1186/1758-2652-13-24)

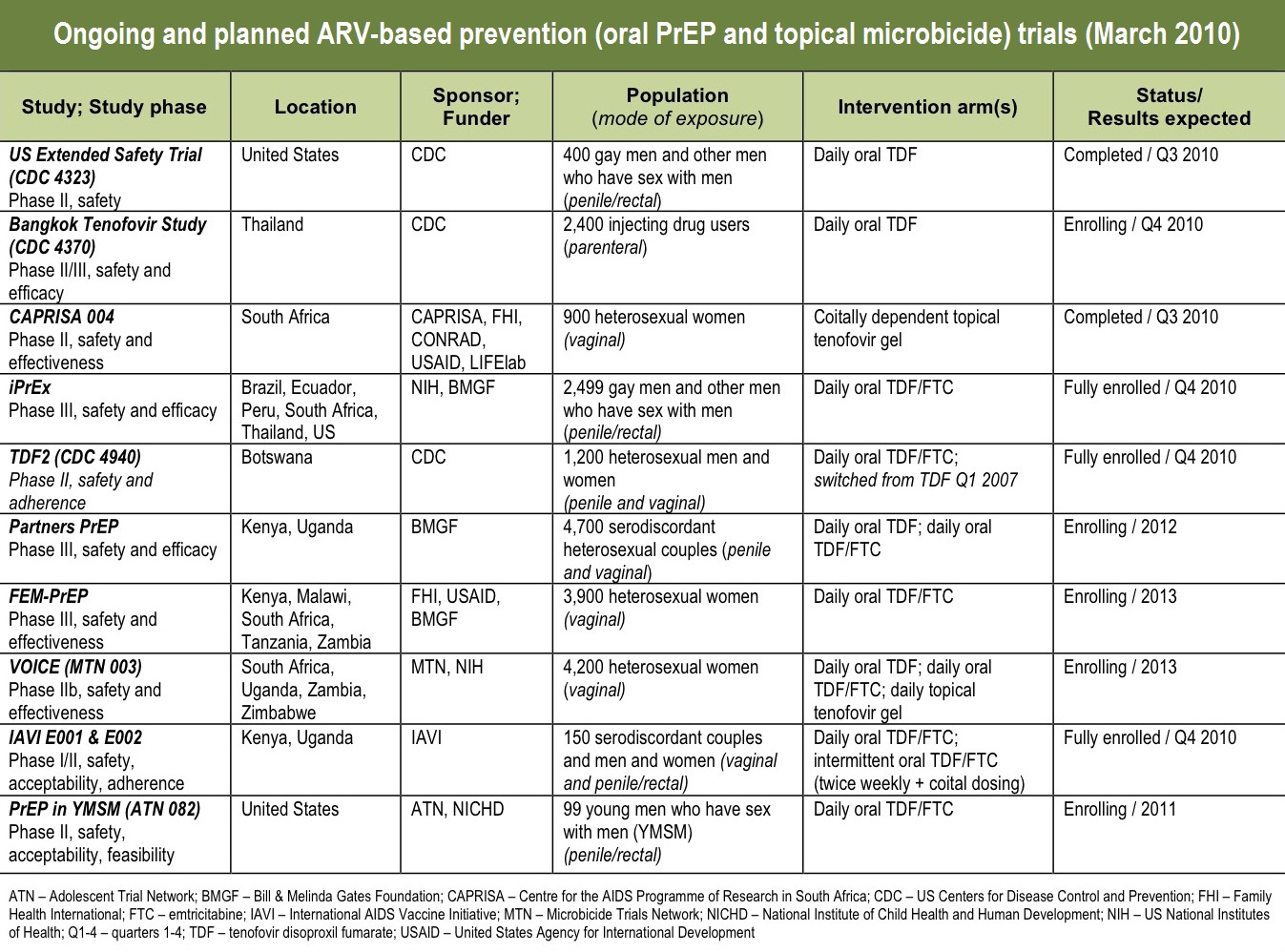

Supplement: Additional file 1 — PrEP trials table - March 2010. Table contains information about ongoing trials of oral PrEP and topical microbicides. (Table: AIDS Vaccine Advocacy Coalition) [file 1758-2652-13-24-S1.JPEG]
